# Rhein Relieves Oxidative Stress in an Aβ_1-42_ Oligomer-Burdened Neuron Model by Activating the SIRT1/PGC-1α-Regulated Mitochondrial Biogenesis

**DOI:** 10.3389/fphar.2021.746711

**Published:** 2021-09-10

**Authors:** Zhihui Yin, Xinyue Geng, Zhengyi Zhang, Ying Wang, Xiaoyan Gao

**Affiliations:** School of Chinese Materia Medica, Beijing University of Chinese Medicine, Beijing, China

**Keywords:** β-amyloid, oxidative stress, rhein, mitochondrial biogenesis, sirtuin 1, peroxisome proliferator-activated receptor gamma coactivator 1-alpha

## Abstract

Neuronal mitochondrial oxidative stress induced by β-amyloid (Aβ) is an early event of Alzheimer’s disease (AD). Emerging evidence has shown that antioxidant therapy represents a promising therapeutic strategy for the treatment of AD. In this study, we investigated the antioxidant activity of rhein against Aβ_1-42_ oligomer-induced mitochondrial oxidative stress in primary neurons and proposed a potential antioxidant pathway involved. The results suggested that rhein significantly reduced reactive oxygen species (ROS) level, reversed the depletion of mitochondrial membrane potential, and protected neurons from oxidative stress-associated apoptosis. Moreover, further study indicated that rhein activated mitochondrial biogenesis accompanied by increased cytochrome C oxidase (CytOx) and superoxide dismutase (SOD) activities. CytOx on the respiratory chain inhibited the production of ROS from electron leakage and SOD helped to eliminate excess ROS. Finally, western blot analysis confirmed that rhein remarkedly increased the protein expression of peroxisome proliferator-activated receptor gamma coactivator 1-alpha (PGC-1α) together with its upstream deacetylase sirtuin 1 (SIRT1), and activated downstream transcription factor nuclear respiratory factor 1, promoting mitochondrial biogenesis. In conclusion, our results demonstrate that rhein activates mitochondrial biogenesis regulated by the SIRT1/PGC-1α pathway as an antioxidant defense system against Aβ_1-42_ oligomer-induced oxidative stress. These findings broaden our knowledge of improving mitochondrial biogenesis as an approach for relieving neuronal oxidative stress in AD.

## Introduction

Alzheimer’s disease (AD), the most common neurodegenerative disease, is characterized by memory loss and cognitive dysfunction (Alzheimer’s Association, 2021). Mounting evidence indicates neuronal oxidative stress induced by β-amyloid (Aβ) is considered to be a critical pathological event in AD, although the etiology of AD has not been fully elucidated (Lin and Beal, 2006; Borger et al., 2011; Swerdlow, 2018). Aβ destroys the electron respiratory chain in the mitochondrial inner membrane by reducing the activities of enzymes in mitochondrial respiratory chain complexes, especially cytochrome C oxidase (CytOx) in complex IV, leading to electron leakage and excess production of reactive oxygen species (ROS) (Atamna and Frey, 2004; Battogtokh et al., 2018; Misrani et al., 2021). Excess ROS aggravates mitochondrial dysfunction, which opens the mitochondrial permeability transition pore (mPTP), accelerates the release of cytochrome c (cyto c) into the cytosol, and subsequently induces the caspase-related apoptosis cascade, triggering the apoptosis of neurons (Mehan et al., 2011; Xiao et al., 2019). Meanwhile, ROS upregulates the expression of amyloid precursor protein and β-site APP-cleaving enzyme 1 by activating the c-Jun N-terminal kinase pathway, facilitates the generation and accumulation of Aβ, and finally accelerates the progression of AD (Chami and Checler, 2012; Tamagno et al., 2012; Zhao and Zhao, 2013; Ganguly et al., 2017). Therefore, relieving oxidative stress is an effective therapeutic strategy for AD.

Mitochondrial biogenesis as a cellular endogenous antioxidant defense system is actively mobilized under pathological conditions to overcome intracellular oxidative stress (Du et al., 2017; Panes et al., 2020). The sirtuin 1 (SIRT1)/peroxisome proliferator-activated receptor-γ coactivator 1-α (PGC-1α) pathway is pivotal for mitochondrial biogenesis (Menzies et al., 2013). SIRT1 is a nicotinamide adenine dinucleotide-dependent deacetylase, which is responsible for deacetylation and activation of the mitochondrial regulatory factor PGC-1α. Activated PGC-1α targets downstream transcription factors, such as nuclear respiratory factor 1 (NRF1) and NRF-2, which upregulate mitochondrial transcription factor A (TFAM). TFAM initiates the transcription and replication of mitochondrial DNA (mtDNA), and ultimately, newly healthy mitochondria are generated for the self-repair of mitochondria and synthesis of antioxidant enzymes (Canto et al., 2009; Kume et al., 2010; Aquilano et al., 2013). The newly generated healthy mitochondria can repair the damaged mitochondrial respiratory chain via mitochondrial fusion through mitochondrial dynamics, inhibiting the production of mitochondria-derived ROS (Liao et al., 2020; Zeng et al., 2021). In addition, synthesis of the mitochondrial antioxidant enzymes, mainly superoxide dismutase (SOD), is increased by generating new mitochondria, thus reducing ROS (St-Pierre et al., 2006; Dhar et al., 2008; Piantadosi and Suliman, 2008; Yan et al., 2016). Unfortunately, under the oxidative stress induced by Aβ, the SIRT1/PGC-1α pathway is suppressed (Gao et al., 2011; Shaerzadeh et al., 2014). Therefore, the activation of SIRT1/PGC-1α-regulated mitochondrial biogenesis as an antioxidant defense system to maintain intracellular mitochondrial homeostasis is essential for relieving the oxidative stress of neurons (Katsouri et al., 2016).

1,8-dihydroxyanthraquinone derivatives are a class of natural compounds with antioxidant activity (Wang et al., 2012; Lu et al., 2015; Markovic et al., 2016; Wang et al., 2016). Representative compounds including rhein, emodin, aloe-emodin, chrysophanol, and physcion are considered candidate compounds for antioxidant therapy. Rhein can protect IEC-6 cells from oxidative damage by inhibiting hydrogen peroxide (H_2_O_2_)-induced ROS, caspase 3, and even apoptosis (Zhuang et al., 2019). Aloe-emodin improves cell viability by reducing the levels of nitric oxide (NO) and ROS in PC12 cells induced by H_2_O_2_ (Tao et al., 2014). Physcion exhibits neuroprotection effect in SH-SY5Y cells by reducing Aβ-induced oxidative stress and the influx of Ca^2+^ (Ho et al., 2015). *In vivo* studies showed that chrysophanol enhanced the activities of SOD and manganese SOD, and inhibited the production of ROS, ultimately relieving oxidative stress injury in the brain of a focal cerebral ischemia/reperfusion mouse model (Zhao et al., 2018). Emodin downregulated 4-hydroxynonenal, a cerebral oxidative damage indicator, increased the expression levels of SOD1 and catalase, and improved the antioxidant capacity of APP/PS1 mice (Li et al., 2021). Therefore, screening of natural compounds with antioxidant activity from anthraquinones is of great clinical significance for relieving oxidative stress and protecting neurons in AD.

As one of the *in vitro* evaluation methods for AD, the cell model is very important for the screening of pharmacodynamic (PD) activity. To date, the screening platform for the evaluation of antioxidants against oxidative stress in AD is still dominated by immortalized cell lines, such as PC12 and SH-SY5Y cells (Lopes et al., 2017; Wang and Xu, 2019; Sotolongo et al., 2020). Nevertheless, after multiple rounds of proliferation and passaging, some traits of immortalized cell lines are quite different from those of living cells *in vivo*. As a consequence, the positive PD results screened from a cell model cannot be replicated when applied to an *in vivo* model. In contrast, the primary neurons obtained from living organisms are closer to the neurons *in vivo*. In addition, recent studies have suggested that Aβ_1-42_ oligomers are the pathogens of early AD (Haass and Selkoe, 2007; Zahs and Ashe, 2013; Fontana et al., 2020). Thus, establishing an Aβ_1-42_ oligomer-induced primary neuron model similar to the pathological environment of AD will greatly improve the screening effectiveness of candidate compounds.

In this study, an AD-like neuronal oxidative stress model was established on primary neurons by optimizing the aggregation states of Aβ_1-42_. Thereafter, the antioxidant activities of five anthraquinones were evaluated by intracellular ROS and mitochondrial membrane potential. As a result, rhein exhibited excellent antioxidant activity and inhibited neuronal apoptosis. Further study was to explore the antioxidant mechanism of rhein. Finally, our results demonstrate that rhein activates mitochondrial biogenesis regulated by the SIRT1/PGC-1α pathway against Aβ_1-42_ oligomer-induced mitochondrial oxidative stress. Taken together, rhein may present a promising candidate molecule for relieving neuronal oxidative stress as well as oxidative stress-associated neurodegenerative disorders.

## Materials and Methods

### Materials and Reagents

Aβ_1-42_ (CAS: 107761-42-2 for lyophilized Aβ_1-42_) was obtained from Nanjing Peptide Biotech Co., Ltd. (Nanjing, China). Rhein (MW 284.22 of purity >98%, CAS: 478-43-3), 1,1,1,3,3,3-hexafluoro-2-propanol (HFIP, CAS: 920-66-1), thioflavin T (ThT, CAS: 2390-54-7), and phosphotungstic acid (CAS: 12501-23-4) were obtained from Shanghai Aladdin Biochemical Technology Co., Ltd. (Shanghai, China). Emodin (MW 270.24 of purity >98%, CAS: 518-82-1), aloe-emodin (MW 270.24 of purity >97%, CAS: 481-72-1), chrysophanol (MW 254.24 of purity >98%, CAS: 481-74-3), and physcion (MW 284.26 of purity >98%, CAS: 521-61-9) were obtained from Shanghai yuanye Bio-Technology Co., Ltd. (Shanghai, China). B27 (Cat: 17504044) and penicillin-streptomycin (PS, Cat: 10378016) were purchased from Gibco Corporation (Grand Island, NY, USA). Dulbecco’s modified Eagle medium (DMEM, Cat: C11995500), Neurobasal medium (Cat: 21103049), and l-glutamine (Cat: A2916801) were purchased from Invitrogen Corporation (Carlsbad, CA, USA). Poly-l-lysine (Cat: P2100), 3-(4,5-dimethylthiazol-2-yl)-2,5-diphenyltetrazolium bromide (MTT, Cat: M8180), and CytOx activity detection kit (Cat: BC0945) were obtained from Beijing Solarbio Science & Technology Co., Ltd. (Beijing, China). Bicinchoninic acid (BCA) protein quantification kit (Cat: P0010), ROS assay kit (Cat: S0033), Mitochondrial membrane potential detection kit (Cat: C2006), SOD activity detection kit (Cat: S0101), Annexin V-FITC apoptosis detection kit (Cat: C1062), and cytosol/mitochondria fraction isolation kit (Cat: C3601) were obtained from Beyotime Biotechnology (Shanghai, China). Primary antibodies, MAP2 rabbit polyclonal antibody (Cat: 17490-1-AP), PGC-1α monoclonal antibody (Cat: 66369-1-Ig), NRF1 polyclonal antibody (Cat: 12482-1-AP), caspase 3 polyclonal antibody (Cat: 19677-1-AP), cyto c polyclonal antibody (Cat: 10993-1-AP), and β-Actin monoclonal antibody (Cat: 66009-1-Ig) were obtained from Proteintech Group, Inc. (Rosemont, IL, USA). SIRT1 monoclonal antibody (Cat: 9475S) was purchased from Cell Signaling Technology Inc. (Danvers, MA, USA). Secondary antibodies, goat anti-mouse IgG (Cat: SA00001-1) and goat anti-rabbit IgG (Cat: SA00001-2) were obtained from Proteintech Group, Inc. All other chemicals used were of the highest grade commercially available.

### Aβ_1-42_ Preparation

Aβ_1-42_ monomers were prepared as described previously (Sinha et al., 2011). The lyophilized Aβ_1-42_ peptides were dissolved in HFIP at a concentration of 1 mg/ml and sonicated for 10 min in an ice-bath, followed by incubation with shaking at 4°C for 3 h. Subsequently, the resulting solution was evaporated under a gentle stream of N_2_ gas to remove HFIP, and peptide films were formed. Thereafter, the peptide films were dissolved in 10 mM sodium hydroxide alone with sonication for 1 min and centrifuged at 4°C (16000 × g, 10 min). Finally, the supernatant was collected and quantified using a NanoDrop One spectrometer (Thermo Fisher Scientific, Waltham, MA, United States) at 280 nm using an extinction coefficient of 1490 M^−1^ cm^−1^.

To prepare different aggregation stages of Aβ_1-42_, Aβ_1-42_ monomers were diluted in phosphate-buffered solution (PBS, 10 mM, pH 7.4) at a final peptide concentration of 50 μM and incubated with continuous orbital shaking at 150 rpm. At different incubation time points, Aβ_1-42_ solution was collected for corresponding experiments.

### Circular Dichroism Spectra Assay

The prepared Aβ_1-42_ was diluted in PBS at a final concentration of 25 μM. 100 μL sample was added to a 0.5 mm path length quartz cell. The spectrum was collected with an average of three scans at a speed of 1 nm/s over the wavelength range from 195 to 260 nm using a Chirascan spectrometer (Applied Photophysics Ltd., Surrey, BA, United Kingdom).

### ThT Fluorescence Assay

The kinetic process of Aβ_1-42_ aggregation was monitored by ThT, the fluorescence intensity of which is dependent on the formation of β-sheet. At different incubation times, Aβ_1-42_ solution was taken for fluorescence assay at the concentration of 1 μM for Aβ_1-42_ and 10 μM for ThT, respectively. The fluorescence was measured using a LS45 fluorescence spectrometer (PerkinElmer Inc., Waltham, MA, United States). The excitation wavelength and emission wavelength were set at 440 and 480 nm, respectively.

### Transmission Electron Microscopy

The prepared Aβ_1-42_ (20 μL) was dropped on a carbon-coated copper grid. After 1 h, the redundant solution was removed with filter paper, and prepared Aβ_1-42_ was stained with 20 μL of 1% phosphotungstic acid for 90 s. Then, the transmission electron microscopy (TEM) images were taken using a JEM-1230 transmission electron microscope (JEOL Ltd., Akishima-shi, Japan) with an acceleration voltage of 100 kV.

### Primary Neuron Cultures

Primary neurons were extracted and isolated from neonatal SD rats as described previously (Dong et al., 2018). Neonatal SD rats were obtained from SPF Biotechnology Co., Ltd. (Beijing, China). Primary neurons were seeding in poly-l-lysine precoated 96-well plates or 24-well plates with a cell density at 5 × 10^5^ cells/mL and cultured in neurobasal medium supplemented with 2% B27 supplements, 1% l-glutamine, and 1% PS at 37°C in a humidified atmosphere containing 5% CO_2_. After culture for 7 days, the primary neurons can be used for experiments.

As illustrated in Supplementary Figure 1, the purity of primary neurons was identified to be above 95% by the immunofluorescence assay labelled with anti-MAP2 antibodies, and the purity of the primary neurons met the requirements for subsequent experiments.

### Cell Treatments

To examine the cytotoxicity of Aβ_1-42_ in different aggregation, primary neurons were incubated with DMEM containing 5 μM Aβ_1-42_ aggregates. After incubation for 24 h, the cell viability was measured. To examine the antioxidant effects of the five anthraquinones against Aβ_1-42_ oligomer-induced oxidative stress, primary neurons which were incubated with DMEM containing 5 μM Aβ_1-42_ and different concentrations of anthraquinones (1, 5 and 10 μM as low, medium, and high doses, respectively) were the treated groups. After incubation for 24 h, treated groups were used for further experiments. Meanwhile, untreated primary neurons were the control group, and primary neurons treated with 5 μM Aβ_1-42_ oligomers alone were the Aβ_1-42_ group.

### Cell Viability Assay

Cell viability was measured by the MTT assay. After the cell treatment, the medium in 96-well plates was discarded. Then, 100 μL DMEM containing MTT at a final concentration of 0.5 mg/ml was added into 96-well plates. After incubation for another 4 h, the solution was discarded followed by adding 100 μL DMSO. Finally, the absorbance at 490 nm was measured using an Epoch Microplate Spectrophotometer (BioTek Instruments Inc., Winooski, VT, United States).

### Intracellular ROS Measurement

ROS in primary neurons was detected using a DCFH-DA probe. DCFH-DA at a concentration of 10 μM was added to 96-well plates. After incubation for 20 min at 37°C, primary neurons were washed three times to fully remove excessive DCFH-DA. Finally, fresh culture medium was added and fluorescence images were taken under a fluorescence microscope (Nikon Instruments Inc., Melville, NY, United States).

### Mitochondrial Membrane Potential (ΔΨm) Measurement

ΔΨm in primary neurons was detected using a JC-1 probe. Mitochondria were stained with a JC-1 staining kit according to the manufacturer’s instructions. Briefly, JC-1 staining solution was added to 24-well plates. After incubation for 20 min at 37°C, primary neurons were washed twice to fully remove excessive JC-1. Finally, fresh culture medium was added and fluorescence images were taken under a fluorescence microscope.

### Molecular Docking

To study the structure-activity relationship of the five anthraquinones, rhein, emodin, aloe-emodin, chrysophanol, and physcion were selected as ligand molecules, and docked with the receptor of SIRT1 (PDB code: 4I5I), respectively. The 3D structures of the five ligand molecules were acquired from the Pubchem Compound database (https://pubchem.ncbi.nlm.nih.gov/). The X-ray crystal structure of human Sirt1 was obtained from the Protein Data Bank (https://www.rcsb.org/). Molecular docking was performed by AutoDock Vina 1.1.2 and AutoDock Tools (The Scripps Research Institute, La Jolla, CA, United States). The size of the grid box in AutoDock Vina was kept as 40 × 40 × 40 for X, Y, and Z. The final model was determined based on the binding energy and molecular interaction. Docked complexes were analyzed and figures were exported using PyMOL 2.3.4 (Schrödinger, Inc., New York, NY, United States).

### Measurement of CytOx Activity

CytOx activity was measured using a CytOx activity detection kit according to the manufacturer’s instructions. Briefly, primary neurons after treatment were washed once with cold PBS and lysed in an extracting solution. The lysate was centrifuged at 4°C (600 × g, 10 min), and the supernatant was transferred to another tube for centrifugation at 4°C (11000 × g, 15 min). Then, the sediment was collected and extracted by extracting solution. After sufficient ultrasonication, each sample was quantified by a BCA protein quantification kit. Finally, 20 μL sample, and 200 μL working solution were added to 96-well plates and the absorbance at 550 nm was measured using an Epoch Microplate Spectrophotometer.

### Measurement of SOD Activity

SOD activity was measured using a SOD activity detection kit according to the manufacturer’s instructions. Briefly, primary neurons after treatment were washed once with cold PBS and lysed in a SOD preparation solution. The lysate was centrifuged at 4°C (16000 × g, 5 min), and the supernatant was collected. Each sample was quantified by a BCA protein quantification kit. Then, 20 μL protein sample, 160 μL working solution, and 20 μL reaction working solution were added to 96-well plates. After incubation for 30 min at 37°C, the absorbance at 450 nm was measured using an Epoch Microplate Spectrophotometer.

### Annexin V-FITC/Propidium Iodide Staining Assay

Apoptosis was evaluated using an Annexin V-FITC apoptosis detection kit according to the manufacturer’s instructions. In brief, primary neurons after treatment were washed once with cold PBS. Then, 195 μL Annexin V-FITC binding buffer, 5 μL Annexin V- FITC, and 10 μL propidium iodide (PI) were sequentially added to 96-well plates. After incubation for 15 min in the dark, fresh culture medium was added and fluorescence images were taken under a fluorescence microscope.

### Western Blot

Primary neurons after treatment were washed once with cold PBS and lysed in an ice-cold lysis buffer containing 20 mM Tris-HCl pH 7.5, 150 mM NaCl, 1% Triton X-100, 1 mM PMSF and other inhibitors including sodium pyrophosphate, β-glycerophosphate, EDTA, Na_3_VO_4_ and leupeptin. The lysate was centrifuged at 4°C (16000 × g, 5 min), and the supernatant was collected. Cytosolic fraction for cytosolic cyto c was prepared using a cytosol/mitochondria fraction isolation kit. After quantification of protein concentration, equivalent amounts of sample were separated by an SDS-PAGE and then transferred onto polyvinylidene fluoride membranes. After being blocked with 5% non-fat milk for 2 h at room temperature, the membranes were incubated with primary antibodies of interest overnight at 4°C and followed by the incubation with secondary antibodies for 2 h at room temperature. The immunoblots were developed with ECL reagents and visualized by a ChemiDoc™ MP Imaging System (Bio-Rad Laboratories, Inc., Hercules, CA, United States). The results were normalized to β-Actin and analyzed using ImageJ software (National Institutes of Health, Bethesda, MD, United States).

### Statistical Analysis

All data were presented as means ± standard deviation (SD) for at least three independent experiments. Statistical significance was analyzed using one-way analysis of variance (ANOVA), followed by the least significant difference (LSD) test for multiple comparisons. Levels of significance were indicated as follows: * *p* < 0.05; ** *p* < 0.01; *** *p* < 0.001. All statistical analyses were performed using SPSS Statistics 25.0 software (IBM Corporation, Armonk, NY, United States).

## Results

### Aβ_1-42_ Oligomer-Induced Oxidative Stress Model Established Based on Primary Neurons

**FIGURE 1:**
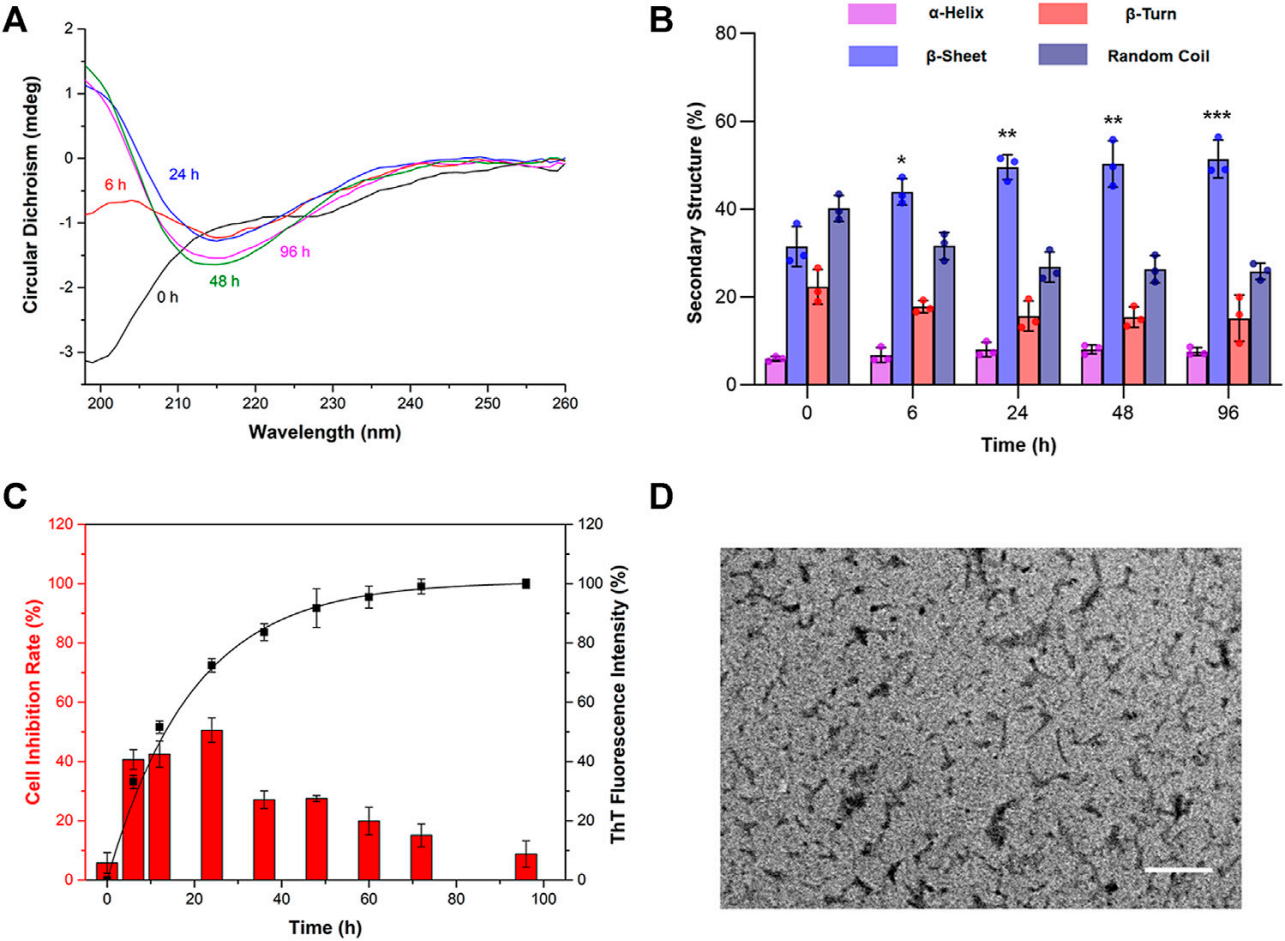
The cytotoxicity of Aβ_1-42_ in different aggregation stages in primary neurons. Aβ_1-42_ monomers were incubated by continuous orbital shaking at 150 rpm and 37°C. **(A)** The changes in the secondary structure of prepared Aβ_1-42_ in different aggregation stages were detected by circular dichroism (CD) spectra. **(B)** The proportions of the secondary structure were further analyzed. **(C)** The aggregation process of Aβ_1-42_ was investigated by the thioflavin T (ThT) assay (*n* = 4) and the cytotoxicity of Aβ_1-42_ in different aggregation stages in primary neurons was determined by the MTT assay (*n* = 5). **(D)** Representative transmission electron microscope image of Aβ_1-42_ incubated for 24 h at 37°C. Scale bars: 200 nm. Data are presented as the mean ± standard deviation (SD). *p < 0.05, **p < 0.01 and ***p < 0.001 compared with the proportions of β-sheets at 0 h (one-way ANOVA).

To establish an *in vitro* model of AD-like oxidative stress, the preparation of Aβ_1-42_ was optimized. First of all, the changes in the secondary structure of prepared Aβ_1-42_ with different incubation times were detected by circular dichroism (CD) spectra. As shown in Figure 1A, Aβ was in a monomeric state at 0 h and was structured as a random coil with a negative absorption peak at 198 nm. With the extension of incubation time, the absorption at 198 nm gradually weakened, and a new absorption peak appeared at 215 nm at 6 h. Its characteristic β-sheet structure at 215 nm revealed that Aβ changed from random coil in the monomer state to β-sheet (i.e., gradually aggregated). With increasing incubation times of 24, 48, and 96 h, the absorption at 215 nm gradually enhanced as well. As shown in Figure 1B, the secondary structure illustrated that the proportions of β-sheets at 6, 24, 48, and 96 h were 32, 44, 50, and 51%, respectively, implying that Aβ gradually aggregated and tended to be stable. The aggregation process of Aβ_1-42_ was further investigated by ThT fluorescence assay. As shown in Figure 1C, the fluorescence intensity of ThT rapidly increased from 6 to 36 h, indicating that Aβ_1-42_ was in the exponential growth phase. After 48 h, the curve grew slowly and gradually reached the plateau phase. These results demonstrated that the prepared Aβ_1-42_ depicted the typical aggregation kinetic process of monomer-oligomer-protofibril-fibril.

To examine the cytotoxicity of Aβ_1-42_ in different aggregation stages in primary neurons, Aβ_1-42_ aggregates prepared at different incubation times in the final concentration of 5 μM were used to evaluate the cell inhibition rate by the MTT assay. The results are shown in Figure 1C. The Aβ_1-42_ monomer did not cause distinct toxicity, and the cell inhibition rate was lower than 10%. With the extension of incubation time, the toxicity of Aβ_1-42_ significantly increased. After incubation for 6, 12, and 24 h, the toxicity of Aβ_1-42_ continued to increase, with inhibition rates of 41, 42, and 51%, respectively. At the plateau phase, the toxicity began to decrease. At 48, 60, 72, and 96 h, the inhibition rates of neurons were 27, 20, 15, and 9%, respectively. According to the abovementioned results, Aβ_1-42_ at 24 h caused maximum damage to the neurons. The morphology of the Aβ_1-42_ aggregates at 24 h was further characterized by TEM. After phosphotungstic acid staining, Aβ_1-42_ had a width of ∼5 nm and a length of <200 nm in the TEM image, suggesting that it was in the oligomeric form (Figure 1D). Taken together, the Aβ_1-42_ oligomers obtained by incubation for 24 h were the most toxic, which could be used to establish an AD oxidative stress cell model.

**FIGURE 2:**
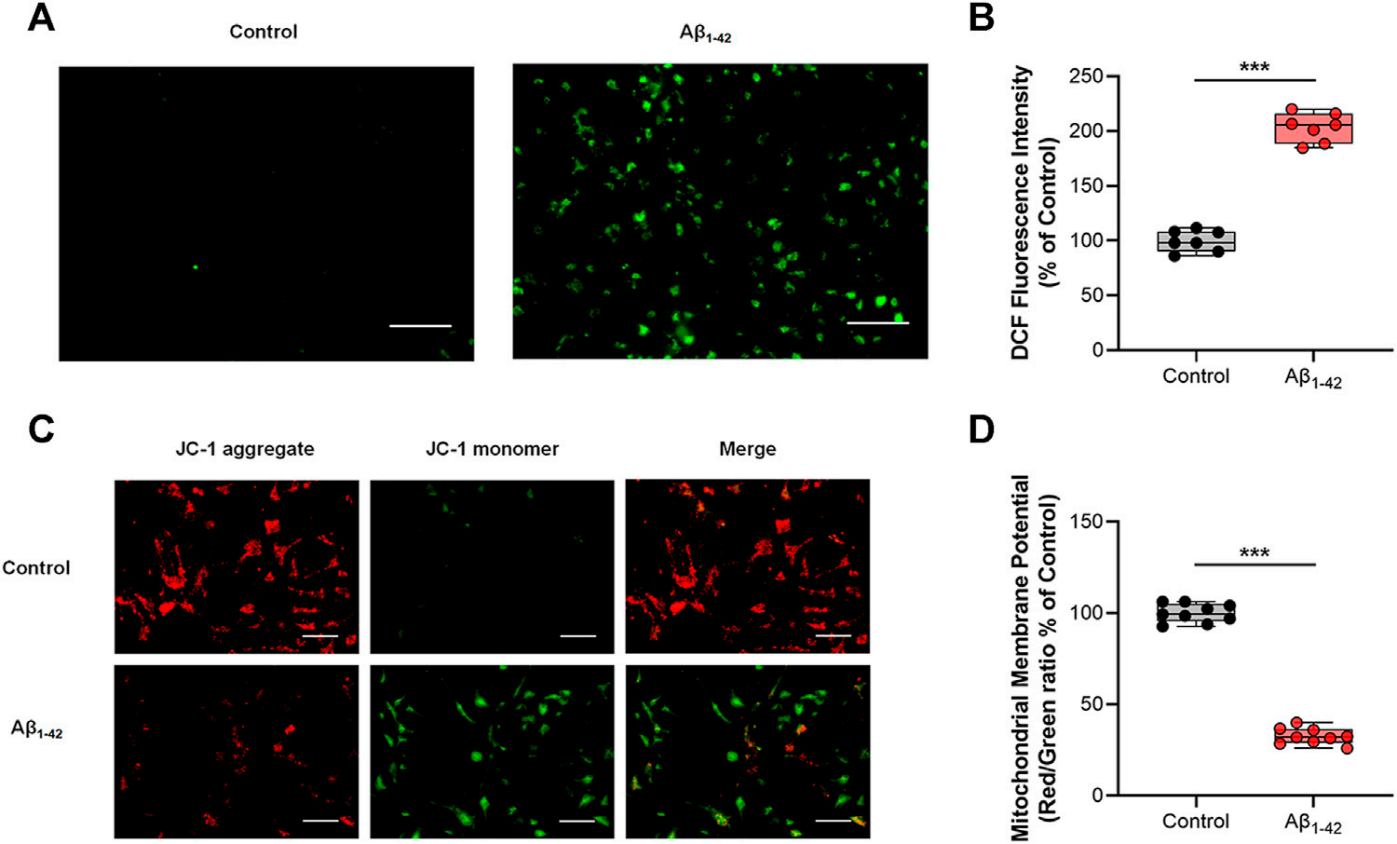
Aβ_1-42_ oligomer-induced oxidative stress model on primary neurons. Untreated primary neurons were the control group, and primary neurons treated with 5 μM Aβ_1-42_ oligomers alone were the Aβ_1-42_ group. **(A)** Representative fluorescence images of intracellular ROS. **(B)** Quantification of intracellular ROS by the fluorescence intensity of DCF and normalization to the control group (*n* = 7). **(C)** Representative fluorescence images of ΔΨm. **(D)** Quantification analysis of ΔΨm by the fluorescence intensity of red/green ratio with JC-1 and normalization to the control group (*n* = 9). Scale bars: 50 μm. Data are presented as the mean ± standard deviation (SD). ***p < 0.001 compared with the Aβ_1-42_ group (one-way ANOVA).

After determining the preparation method for toxic Aβ, primary neurons were stimulated by Aβ_1-42_ oligomers to induce oxidative stress. The intracellular ROS level was detected by the DCFH-DA probe. After being oxidized by ROS, DCFH-DA transformed to DCF, a fluorescent substance, and the fluorescence intensity of DCF was used to evaluate intracellular oxidative stress. As illustrated in Figures 2A,B, compared with the control group, the fluorescence intensity of DCF in the Aβ_1-42_ group increased to 203%, indicating that Aβ_1-42_ oligomers induced high ROS levels in primary neurons. Because intracellular ROS was derived from mitochondrial dysfunction, the intracellular ΔΨm was detected with a JC-1 probe to evaluate mitochondrial function. As shown in Figures 2C,D, the Aβ_1-42_ group exhibited more JC-1 monomers (green fluorescence) and fewer JC-1 aggregates (red fluorescence) than the control group. The fluorescence intensity of the red/green ratio decreased to 32% after being normalized to the control group, suggesting that a lower ΔΨm was due to mitochondrial damage. These results demonstrated that Aβ_1-42_ oligomers damaged mitochondrial function and caused oxidative stress in primary neurons.

### Evaluation of the Antioxidative Activities of the Five Anthraquinones in Alleviating Aβ_1-42_ Oligomer-Induced Oxidative Stress in Primary Neurons

**FIGURE 3:**
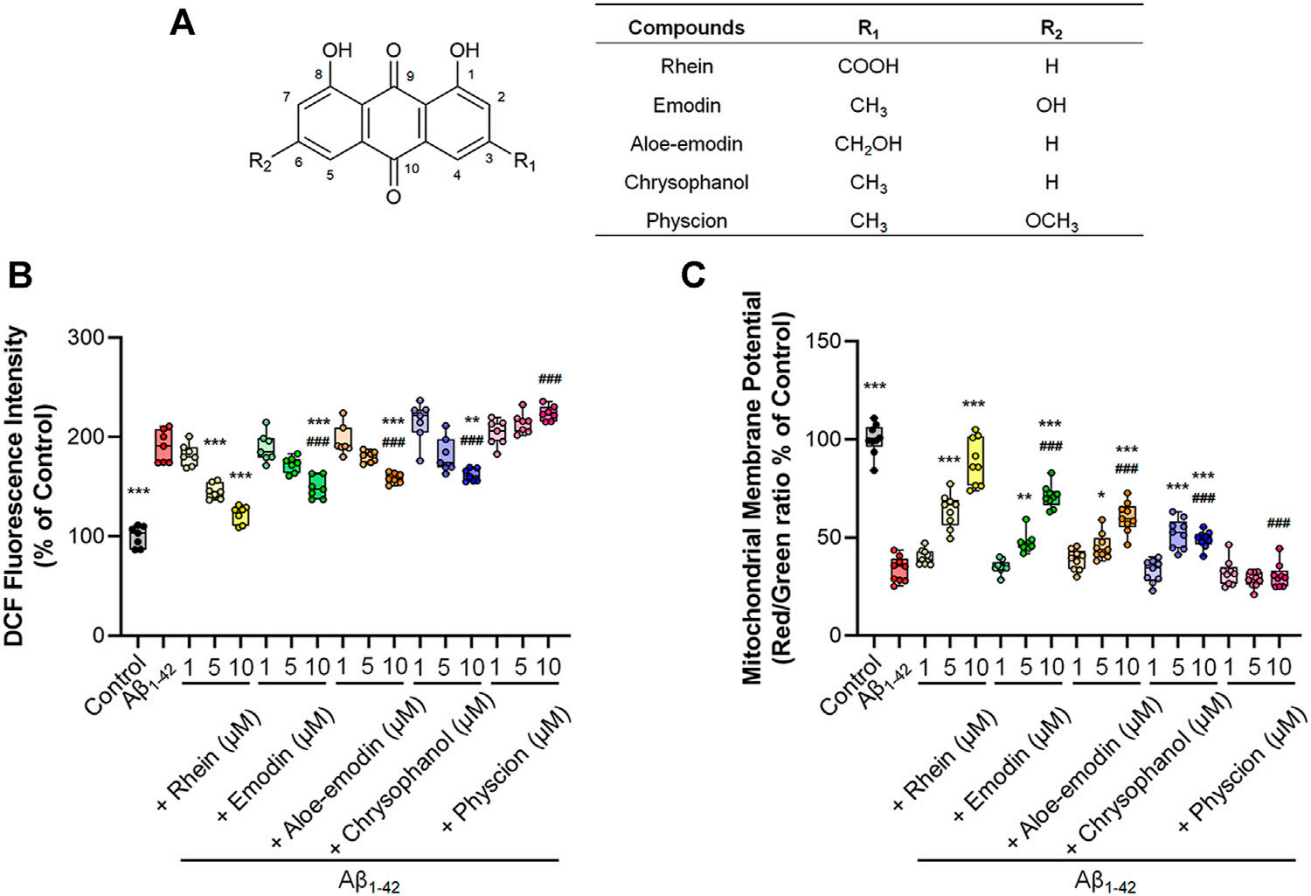
The antioxidant effects of the five anthraquinones on primary neurons. Primary neurons were incubated with 5 μM Aβ_1-42_ oligomers and different concentrations of anthraquinones (1, 5 and 10 μM) for 24 h at 37°C, respectively. Untreated primary neurons were the control group, and primary neurons treated with 5 μM Aβ_1-42_ oligomers alone were the Aβ_1-42_ group. **(A)** The chemical structures of rhein, emodin, aloe-emodin, chrysophanol, and physcion. **(B)** Quantification of intracellular ROS by the fluorescence intensity of DCF and normalization to the control group (*n* = 7). **(C)** Quantification analysis of ΔΨm by the fluorescence intensity of red/green ratio with JC-1 and normalization to the control group (*n* = 9). Data are presented as the mean ± standard deviation (SD). *p < 0.05, **p < 0.01, ***p < 0.001 compared with the Aβ_1-42_ group; ###*p* < 0.001, compared with the 10 μM rhein group (one-way ANOVA).

After establishing an oxidative stress neuron model induced by Aβ_1-42_ oligomers, we used this model to investigate the antioxidant activities of five anthraquinones. The chemical structures of the five anthraquinones are displayed in Figure 3A. They shared 1,8-dihydroxy anthraquinone as the same structure, yet they were substituted with different functional groups at the 3- or 6-position. Thereafter, the effects of the five anthraquinones on intracellular ROS level were determined. As shown in Figure 3B and Supplementary Figure 2, compared with the Aβ_1-42_ group, rhein treatment at doses of 1, 5, and 10 μM inhibited intracellular ROS levels by 182, 145, and 122%, respectively, manifesting an obvious dose dependency. The 5 μM rhein was sufficient to produce a significant difference, while the 10 μM rhein reduced ROS to the level of the control group. Emodin was also able to lower intracellular ROS levels. Emodin treatment at doses of 1, 5, and 10 μM reduced ROS levels by 189, 172, and 149%, respectively. Nevertheless, a significant difference was only found in the 10 μM emodin group. Aloe-emodin treatment at doses of 1, 5, and 10 μM reduced intracellular ROS levels by 196, 180, and 159%, respectively. Similar to aloe-emodin, the ROS scavenging effects of chrysophanol at doses of 1, 5, and 10 μM were 215, 181, and 162%, respectively. However, none of the three doses of physcion were able to effectively reduce intracellular ROS levels. The abovementioned results manifested that rhein, emodin, aloe-emodin, and chrysophanol could alleviate intracellular oxidative stress to varying degrees, with the exception of physcion.

The difference in antioxidant activity among the five anthraquinones was analyzed under the same condition, by comparing emodin, aloe-emodin, chrysophanol, and physcion with rhein at the same dose of 10 μM. Statistical analyses and the tendency of ROS to alleviate oxidative stress showed that the comparison for antioxidant activities of the five anthraquinones was rhein > emodin > aloe-emodin > chrysophanol > physcion.

JC-1 was used as an indicator to detect the intracellular ΔΨm. Figure 3C and Supplementary Figure 3 illustrated the results of ΔΨm. With the exception of physcion, which did not alleviate oxidative stress, the other four anthraquinones restored ΔΨm to varying degrees. The 1 μM rhein slightly increased the ΔΨm and the difference was insignificant. The 10 μM rhein increased the ΔΨm to 89%, and different doses presented a distinct dose-dependent increase. The effects of emodin and aloe-emodin were similar, also manifesting a dose-dependent increase with the strongest effects occurring at the dose of 10 μM (71 and 60%, respectively). The effect of the 5 μM chrysophanol on ΔΨm was stronger than that of 1 μM but was not significantly different from that of 10 μM.

Likewise, the intensity difference of the recovery effect among the five anthraquinones on ΔΨm was analyzed by comparing emodin, aloe-emodin, chrysophanol, and physcion with rhein at the same dose of 10 μM. Statistical analyses showed that the comparison for the effects of the five anthraquinones was rhein > emodin > aloe-emodin > chrysophanol > physcion.

### Molecular Docking Simulation and Structure-activity Relationship Analysis

SIRT1-dependent deacetylation of PGC-1α promotes mitochondrial biogenesis, which can repair the function of the neuronal mitochondrial respiratory chain and increase the level of antioxidant enzymes, thereby synergically alleviating Aβ-induced oxidative stress (Lagouge et al., 2006; Li et al., 2020; Lu et al., 2020). Under the oxidative stress in AD, the expression levels of both SIRT1 and PGC-1α are reduced, leading to impaired mitochondrial biogenesis. Hence, oxidative stress in neurons cannot be alleviated (Qin et al., 2009; Sheng et al., 2012). Here, to compare the impact of the structural differences of the five anthraquinones on their antioxidant activity, SIRT1 was selected as the potential receptor protein to study the structure-activity relationship by molecular docking.

**FIGURE 4:**
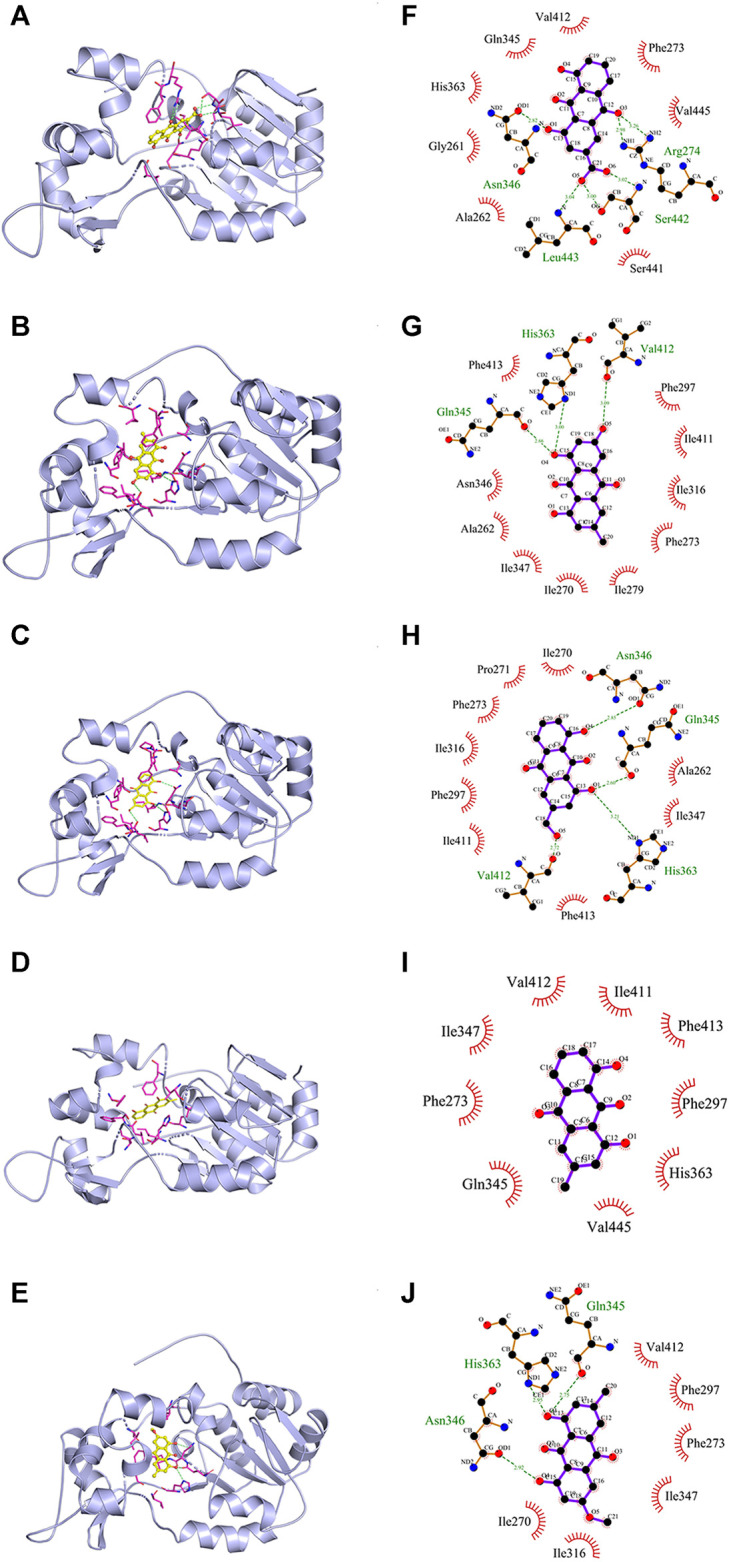
The molecular simulation between five anthraquinones and SIRT1 (PDB code: 4I5I). **(A-E)** Rhein, emodin, aloe-emodin, chrysophanol, and physcion in the active site of SIRT1. **(F-J)** The binding modes of the five ligands with receptor SIRT1. Green dotted line: H-bonds; red arc: hydrophobic interaction.

First of all, SIRT1 was taken as the receptor protein (PDB code: 4I5I) to analyze its binding capacity to anthraquinones. As shown in Figures 4A–E, all the five anthraquinones were directly bound to SIRT1 and occupied its active site, indicating that anthraquinones could interact with SIRT1. As shown in Table 1, the binding energy between rhein, emodin, aloe-emodin, chrysophanol, and physcion and SIRT1 was −9.4, −9.1, −8.8, −9.0, and −8.8 kcal/mol, respectively. Among them, the binding capacity between rhein and SIRT1 was the strongest, followed by emodin and chrysophanol, while the binding capacity of aloe-emodin was relatively weaker and equivalent to that of physcion.

**TABLE 1:** Binding affinity of the five anthraquinones with SIRT1.

Ligands	Binding energy (kcal/mol)	H-bonds		Hydrophobic interaction	
		Number	Interacting residues	Number	Interacting residues
Rhein	−9.4	6	Arg274, Ser442, Asn346, Leu443	8	His363, Gly261, Ala262, Ser441, Val445, Phe273, Val412, Gln345
Emodin	−9.1	3	His363, Gln345, Val412	10	Phe413, Phe297, Ile411, Ile316, Phe273, Ile279, Ile270, Ile347, Ala262, Asn346
Aloe-emodin	−8.8	4	Asn346, Gln345, His363, Val412	9	Ile270, Pro271, Phe273, Ile316, Phe297, Ile411, Phe413, Ile347, Ala262
Chrysophanol	−9.0	—	—	9	Val412, Ile347, Phe273, Gln345, Val445, His363, Phe297, Phe413, Ile411
Physcion	−8.8	3	Gln345, His363, Asn346	6	Val412, Phe297, Phe273, Ile347, Ile316, Ile270

Subsequently, the effect of substituents of anthraquinones on the binding capacity to SIRT1 was further compared by analyzing the binding mode. As shown in Figures 4F–J and Table 1, hydrogen bonds and hydrophobic interactions existed between SIRT1 and the five anthraquinones, which jointly determined the binding stability of the receptor-ligand. In terms of hydrophobicity, since the five anthraquinones shared the same hydrophobic structure of 1,8-dihydroxyanthraquinone, they all formed corresponding hydrophobic interactions with different amino acid residues on SIRT1, and different substituents had little effect on the number of residues of hydrophobic interactions. In contrast, the substituents had a noticeable effect on the number of hydrogen bonds. Rhein formed six hydrogen bonds with residues Arg274, Ser442, Asn346, and Leu443 in the active site of SIRT1. Among them, the carboxyl group at position-3 contributed three hydrogen bonds. Emodin formed three hydrogen bonds with His363, Gln345, and Val412, and the phenolic hydroxyl group at position-6 contributed one hydrogen bond. Aloe-emodin formed four hydrogen bonds with Asn346, Gln345, His363, and Val412. Among them, the hydroxymethyl group at position-3 contributed one hydrogen bond. Due to the lack of hydrogen bond donor or acceptor on the methyl group at position-3, chrysophanol did not form a stable hydrogen bond with SIRT1. Thus, chrysophanol was bound to SIRT1 only through the hydrophobic interactions. The electron distribution in the oxygen atom environment of methoxyl group at position-6 in physcion was in the homogeneous distribution, which impeded the formation of hydrogen bonds with residues in the active site; so, it did not contribute any hydrogen bonds.

The abovementioned results suggested that the polar substituents of anthraquinones (i.e., carboxyl, phenolic hydroxyl, and hydroxymethyl) were dominant groups and enhanced the binding capacity or binding stability with SIRT1. This finding was consistent with the experimental results on antioxidant activity.

### Rhein Inhibited Cell Apoptosis Induced by Mitochondrial Oxidative Stress

**FIGURE 5:**
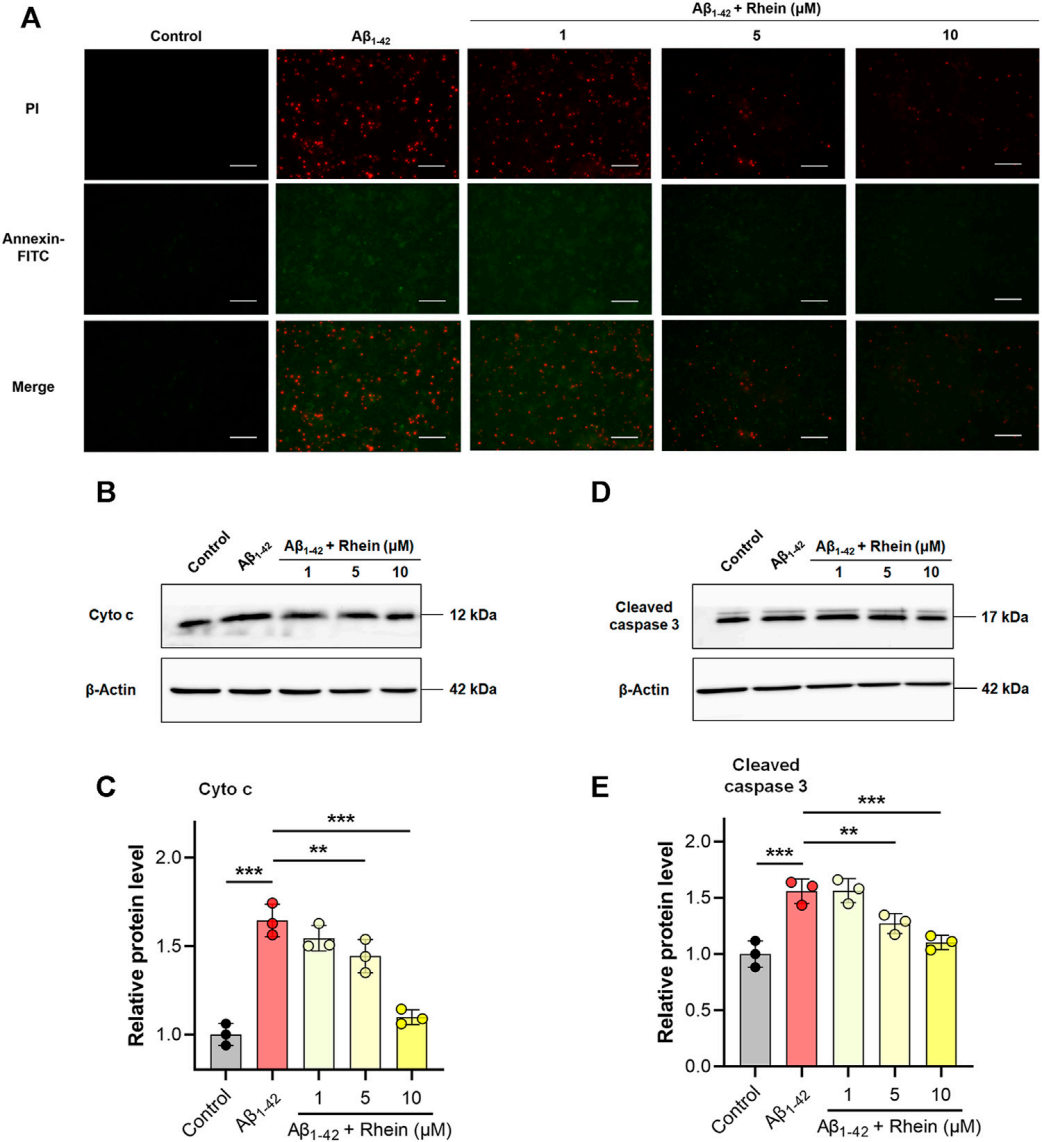
Rhein inhibited mitochondrial oxidative stress-associated apoptosis in primary neurons induced by Aβ_1-42_ oligomers. Primary neurons were incubated with 5 μM Aβ_1-42_ oligomers and rhein at different doses (1, 5 and 10 μM) for 24 h at 37°C, respectively. Untreated primary neurons were the control group, and primary neurons treated with 5 μM Aβ_1-42_ oligomers alone were the Aβ_1-42_ group. **(A)** Representative images of Annexin V-FITC/propidium iodide (PI) double staining. Annexin V-FITC stains early apoptotic cells; PI stains late apoptotic cells. Scale bars: 50 μm. **(B)** Representative western blot images of cytosolic cytochrome c (cyto c). **(C)** Relative expression level of cytosolic Cyto c and normalization to β-Actin (*n* = 3). **(D)** Representative western blot images of cleaved caspase 3. **(E)** Relative expression level of cleaved caspase 3 and normalization to β-Actin (*n* = 3). Data are presented as the mean ± standard deviation (SD). **p < 0.01 and ***p < 0.001 compared with the Aβ_1-42_ group (one-way ANOVA).

Mitochondrial oxidative stress can induce neuronal oxidative damage and apoptosis. Encouraged by the excellent antioxidant activity of rhein, we further confirmed the role of rhein in neuroprotection by alleviating mitochondrial oxidative stress. Annexin V-FITC/PI double staining assay was performed to evaluate the effect of rhein on neuronal apoptosis. As shown in Figure 5A, compared with the control group, the green and red fluorescence intensity in the Aβ_1-42_ group was greatly enhanced, indicating that the neuronal cells were undergoing early and late apoptosis, respectively. Interestingly, compared with the Aβ_1-42_ group, rhein reversed apoptosis. The 10 μM rhein significantly reduced the intracellular green and red fluorescence intensity. The abovementioned results proved that rhein could inhibit the apoptosis of primary neurons by relieving oxidative stress.

Oxidative stress can damage the ultrastructural integrity of mitochondria. Cyto c, an apoptotic factor, is released from the inner mitochondrial membrane to activate procaspase 3 into cleaved caspase 3, which induces the apoptosis cascade. This is a critical pathological process for the loss of neuronal synapses (Ye et al., 2013). To investigate the mechanism underlying anti-apoptosis by rhein, the expression levels of cyto c in the cytosol and cleaved caspase 3 in primary neurons were evaluated by western blotting. The results are shown in Figures 5B,C. Because Aβ_1-42_ induced oxidative damage in mitochondria, the expression of cytosolic cyto c in the Aβ_1-42_ group significantly increased. Impressively, after treatment with rhein, the level of overexpressed cytosolic cyto c decreased, indicating that rhein reduced the release of cyto c from mitochondria. The expression level of cleaved caspase 3 is shown in Figures 5D,E. Compared with the control group, the level of cleaved caspase 3 in the Aβ_1-42_ group was significantly increased, suggesting that the apoptosis cascade was activated. By contrast, rhein inhibited the level of overexpressed cleaved caspase 3. As shown by the above results, rhein inhibited the release of cyto c from mitochondria by relieving oxidative damage in mitochondria to prevent the activation of the apoptosis cascade and ultimately avoid neuronal apoptosis.

### Rhein Increased the Activities of Mitochondrial CytOx and SOD

**FIGURE 6:**
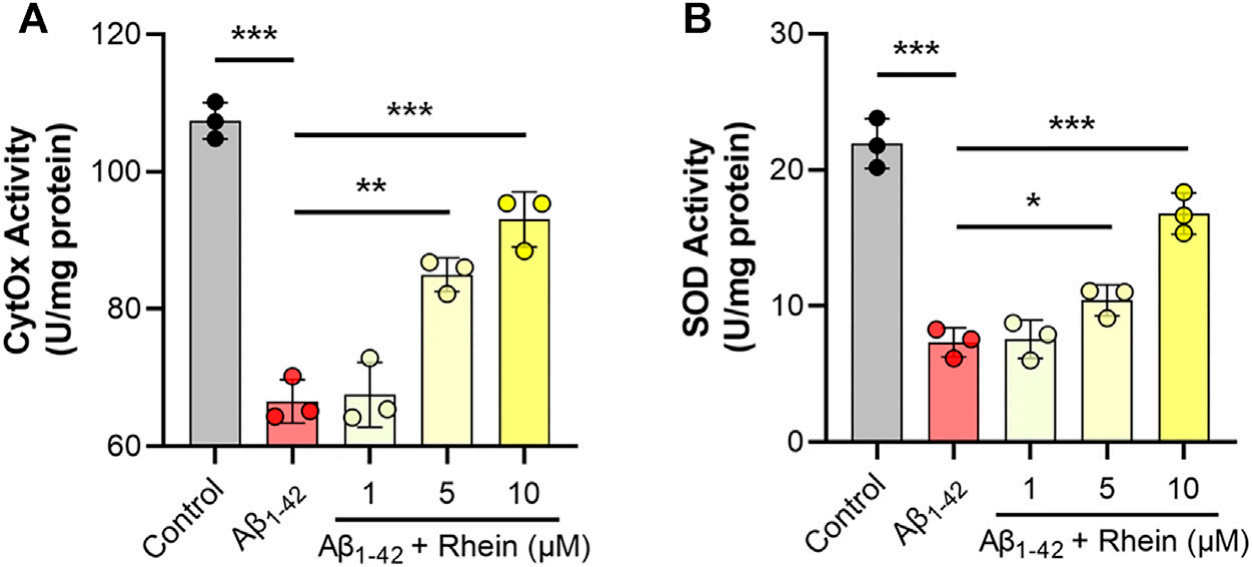
Rhein increased the activities of mitochondrial cytochrome C oxidase (CytOx) and superoxide dismutase (SOD). Primary neurons were incubated with 5 μM Aβ_1-42_ oligomers and rhein at different doses (1, 5 and 10 μM) for 24 h at 37°C, respectively. Untreated primary neurons were the control group, and primary neurons treated with 5 μM Aβ_1-42_ oligomers alone were the Aβ_1-42_ group. The effects of rhein on the activities of **(A)** CytOx and **(B)** SOD (*n* = 3). Data are presented as the mean ± standard deviation (SD). *p < 0.05, **p < 0.01 and ***p < 0.001 compared with the Aβ_1-42_ group (one-way ANOVA).

Considering that oxidative stress can be effectively alleviated through mitochondrial biogenesis, we focused on the effects of rhein on enzymes related to mitochondrial biogenesis, such as CytOx in the mitochondrial respiratory complex IV and antioxidant enzyme SOD. First, the activity of CytOx was evaluated. As shown in Figure 6A, compared with the control group, Aβ_1-42_ damaged the mitochondrial electron transport chain and diminished the activity of CytOx. After treatment with rhein, the activity of CytOx increased. The effect of the 10 μM rhein group was close to that of the control group. Then, the antioxidant capacity of mitochondria was evaluated by detecting SOD activity, a mitochondria-related antioxidant enzyme. As shown in Figure 6B, the activity of SOD decreased in the Aβ_1-42_ group; thus, the antioxidant capacity of primary neurons was reduced as well. Notably, rhein improved the activity of SOD. These results revealed that rhein not only repaired the mitochondrial electron transport chain to inhibit the production of ROS but also improved the activity of mitochondrial antioxidant enzymes by activating the mitochondrial antioxidant defense system, thus enhancing the elimination of intracellular ROS, which might be related to the improvement of mitochondrial biogenesis.

### Rhein Improved Mitochondrial Biogenesis by Activating the SIRT1/PGC-1α Pathway

**FIGURE 7:**
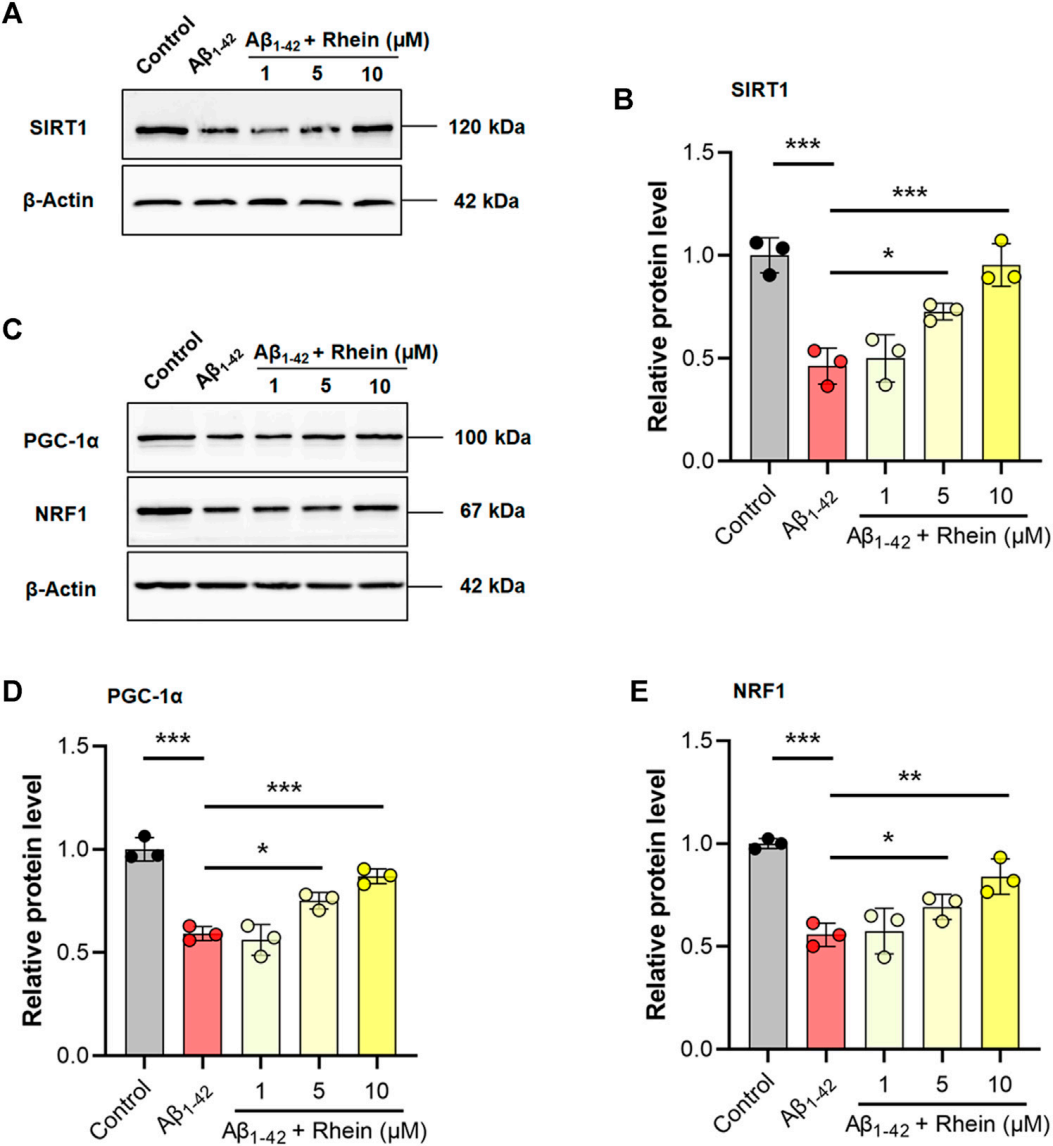
Rhein enhanced mitochondrial biogenesis by activating the SIRT1/PGC-1α pathway. Primary neurons were incubated with 5 μM Aβ_1-42_ oligomers and rhein at different doses (1, 5 and 10 μM) for 24 h at 37°C, respectively. Untreated primary neurons were the control group, and primary neurons treated with 5 μM Aβ_1-42_ oligomers alone were the Aβ_1-42_ group. **(A)** Representative western blot images of SIRT1. **(B)** Relative expression level of SIRT1 and normalization to β-Actin (*n* = 3). **(C)** Representative western blotting images of PGC-1α and NRF1. **(D)** Relative expression level of PGC-1α and normalization to β-Actin (*n* = 3). **(E)** Relative expression level of NRF1 and normalization to β-Actin (*n* = 3). Data are presented as the group mean ± standard deviation (SD). *p < 0.05, **p < 0.01 and ***p < 0.001 compared with the Aβ_1-42_ group (one-way ANOVA).

The SIRT1/PGC-1α pathway is critical for mitochondrial biogenesis. To further validate the regulatory mechanism of rhein for mitochondrial biogenesis, the expression levels of SIRT1 and PGC-1α were detected by western blotting. Compared with the control group, the expression level of SIRT1 was significantly decreased in the Aβ_1-42_ group. As expected, rhein effectively reversed the decreased expression of SIRT1 (Figures 7A,B). Also, Aβ_1-42_ reduced the expression of PGC-1α, indicating that SIRT1/PGC-1α was inhibited in the Aβ-burdened neuronal model (Figures 7C,D). After treatment with rhein, the expression level of PGC-1α was significantly increased. Hence, rhein was able to activate the SIRT1/PGC-1α pathway. In addition, the expression level of NRF1, a downstream transcription factor related to mitochondrial biogenesis, was also detected. The results are shown in Figures 7C,E. In the Aβ_1-42_ group, because of the dysfunction of mitochondrial biogenesis, the expression level of NRF1 decreased. Rhein induced the expression of NRF1 by activating the SIRT1/PGC-1α pathway. It could be concluded that rhein could restore the function of mitochondrial biogenesis to alleviate oxidative stress in primary neurons by increasing the expression of SIRT1, PGC-1α, and NRF1.

## Discussion

Neuronal oxidative stress induced by Aβ is an important pathological feature of AD. High ROS level derived from mitochondrial induces neuronal apoptosis (Lustbader et al., 2004; Petersen et al., 2008). Meanwhile, ROS accelerates the progression of AD by increasing the production and accumulation of Aβ (Takuma et al., 2009; Borger et al., 2013). Thus, introducing antioxidants to relieve oxidative stress is an effective therapeutic strategy for AD. 1,8-dihydroxyanthraquinone derivatives including rhein, emodin, aloe-emodin, chrysophanol, and physcion have garnered widespread attention as antioxidative components. Thus, screening of antioxidants from anthraquinones is extremely promising for the antioxidant therapy of AD. Herein, a comparative analysis of the antioxidant activity of the five anthraquinones was carried out using an Aβ_1-42_ oligomer-induced oxidative stress model based on primary cultured neurons. Among all the five anthraquinones, rhein possessed excellent antioxidant activity and was selected to explore its mechanism of alleviating oxidative stress.

With the progression of AD, accumulated Aβ monomers gradually aggregate and form oligomers, protofibrils, fibrils, and finally senile plaques (Kung, 2012; Wang et al., 2015). The latest β-amyloid cascade hypothesis suggests that Aβ in the oligomeric form is the earliest culprit of AD (Lacor et al., 2004; Staderini et al., 2015; Lee et al., 2017). To establish a drug screening platform that is similar to the *in vivo* pathological environment of oxidative stress in AD, the preparation of toxic Aβ was examined firstly on primary neurons. By combining CD with the ThT assay, the kinetic process of Aβ aggregation was described with β-sheets as the characteristic structure. Then, the correlation between the aggregation form of Aβ and cytotoxicity was analyzed. The results indicated Aβ oligomers incubated for 24 h caused the most damage to the primary neurons, while Aβ monomers or fibrils prepared *in vitro* were less toxic. TEM analysis further confirmed the formation of Aβ oligomers. These findings are consistent with recent researches (Wang-Dietrich et al., 2013; Shea et al., 2019). After incubation with Aβ_1-42_ oligomers, the primary neurons exhibited high ROS level and depletion of ΔΨm, verifying that a mitochondrial oxidative stress cell model was successfully established.

For comparative analysis of antioxidant activities of five anthraquinones, we used aforementioned Aβ_1-42_ oligomer-induced primary neurons as an *in vitro* oxidative stress model. The results suggested that rhein, emodin, aloe-emodin, and chrysophanol reduced intracellular ROS levels to varying degrees in a dose-dependent manner, with the exception of physcion. As expected, rhein, emodin, aloe-emodin, and chrysophanol effectively restored ΔΨm, except physcion. These results indicate that the comparison for antioxidant activities of the five anthraquinones is: rhein > emodin > aloe-emodin > chrysophanol > physcion. To theoretically elucidate the effects of the structural differences of the five anthraquinones on the antioxidant activity, molecular docking was performed to compare the binding mode between anthraquinones and the receptor protein SIRT1. The results showed that rhein, emodin, aloe-emodin, chrysophanol, and physcion were directly bound to the active site of SIRT1. Among them, rhein showed the relatively lower binding energy of −9.4, indicating a better binding capacity with SIRT1. Furthermore, the binding mode showed that the carboxyl group on rhein formed more hydrogen bonds with amino acid residues and increased the stability of the rhein-SIRT1 complex. These findings might partly explain why rhein exhibited excellent antioxidant activity against oxidative stress induced by Aβ_1-42_ oligomers.

Ongoing oxidative stress can trigger apoptosis cascade and induce neuronal apoptosis. When neuronal mitochondria undergo oxidative damage, the apoptosis factor, cyto c, is released from the opened mPTP into the cytosol. Then, procaspase 3 is activated into cleaved caspase 3 by cyto c, which induces the downstream apoptosis cascade and neuronal apoptosis irreversibly (Bonda et al., 2014). Annexin V-FITC/PI staining showed that the number of neuronal cells with early apoptosis (green fluorescence) and late apoptosis (red fluorescence) was significantly increased in the Aβ_1-42_ group, indicating neuronal apoptosis occurred (Figure 5A). After treatment with rhein, the number of apoptotic neurons was effectively reduced. According to the western blot analysis, there was the overexpression of cytosolic cyto c and cleaved caspase 3 in the Aβ_1-42_ group. Interestingly, the levels of cytosolic cyto c and cleaved caspase 3 were reversed after rhein treatment. Thus, rhein reduced the release of cyto c from mitochondria, inhibiting the apoptosis cascade and ultimately protecting neurons from apoptosis.

Neuronal oxidative stress is closely related to mitochondrial dysfunction. Mitochondrial biogenesis is an intracellular antioxidative defense system, in which cells maintain healthy mitochondrial function by producing new mitochondria. Newly generated mitochondria repair the respiratory chain complexes by mitochondrial fusion and further prevent electron leakage, thereby inhibiting the continuous production of ROS. In addition, the increased synthesis of antioxidant enzymes in mitochondria can effectively remove the accumulated ROS to maintain mitochondrial redox homeostasis (Venditti et al., 2013). Under the oxidative stress induced by Aβ, mitochondrial biogenesis is blocked. The activities of enzymes in mitochondrial respiratory chain complexes and antioxidant enzymes decrease, and the antioxidant defense system is also destroyed. Indeed, in this study, it was found that the activity of CytOx, an enzyme in respiratory chain complex IV, was significantly reduced in the mitochondria of primary neurons damaged by Aβ_1-42_ oligomers (Atamna and Frey, 2004; Atamna et al., 2009). However, the activity of CytOx increased after treatment with rhein, indicating that the activity of enzymes in the respiratory chain was improved, which helped to reduce ROS production. Due to the impairment of mitochondrial function caused by Aβ_1-42_ oligomers, the activity of SOD, the main enzyme of mitochondrial antioxidants, was greatly reduced, making mitochondria unable to resist the existing oxidative stress. By contrast, SOD activity was significantly increased under the treatment of rhein, so that mitochondria could effectively remove the existing ROS. The results showed that rhein played a positive role in regulating both the enzymes in mitochondrial respiratory chain complexes and antioxidant enzymes, suggesting that it may be beneficial to improve mitochondrial biogenesis.

**FIGURE 8:**
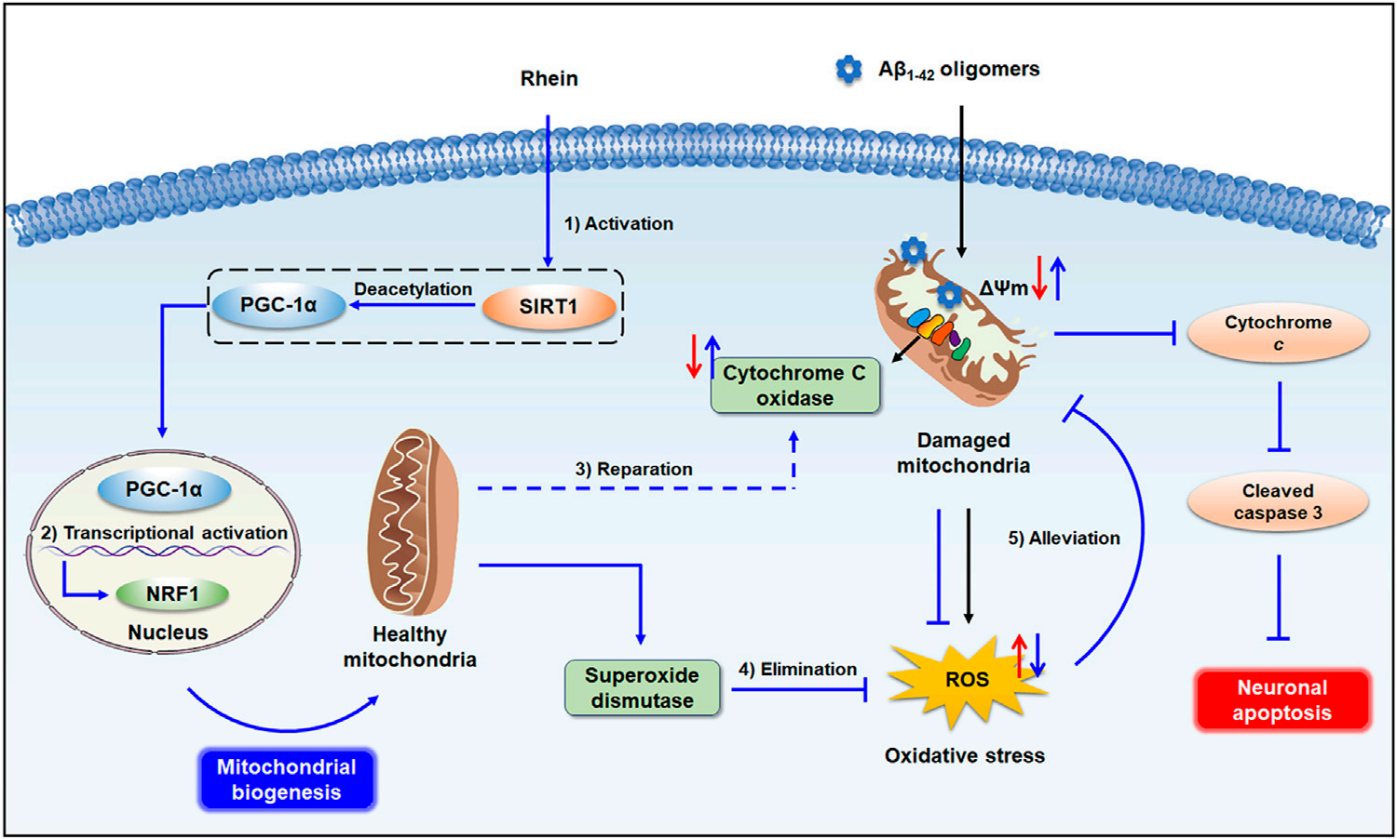
Schematic illustration of mitochondrial endogenous antioxidant defense system triggered by rhein. 1). Rhein activated the SIRT1/PGC-1α pathway by increasing the expression levels of SIRT1 and PGC-1α. 2). The PGC-1α in the nucleus activated the downstream transcription factor NRF1, and healthy mitochondria were generated by promoted mitochondrial biogenesis. 3). Newly generated healthy mitochondria restored the decreased activity of CytOx in mitochondrial respiratory chain complex IV induced by Aβ_1-42_ oligomers, inhibiting the production of ROS. 4). mitochondrial SOD effectively facilitated the elimination of excess ROS. 5). Rhein alleviated the mitochondrial oxidative damage, inhibited the caspase 3-related apoptosis cascade by reducing the release of cyto c from mitochondria, and ultimately, protected neurons from apoptosis.

The SIRT1/PGC-1α pathway plays a crucial role in mitochondrial biogenesis. SIRT1, a deacetylase, is responsible for the deacetylation and activation of PGC-1α. PGC-1α, an important regulator of mitochondrial biogenesis, activates the downstream transcription factors NRF-1, NRF-2, and TFAM, and initiates the transcription and replication of mtDNA. Therefore, the expression of SIRT1 and PGC-1α is closely related to the self-repair of mitochondria and synthesis of antioxidant enzymes (St-Pierre et al., 2006; Kang et al., 2017). To explore the mechanism underlying the activation of mitochondrial biogenesis by rhein, the expression of related proteins was evaluated by western blot analysis. The results confirmed that mitochondrial biogenesis was impaired by Aβ_1-42_ oligomers accompanied by the reduced expression levels of SIRT1, PGC-1α, and downstream transcription factor NRF1. As a result, after treatment with rhein, the expression levels of SIRT1 and PGC-1α were significantly increased, indicating that the SIRT1/PGC-1α pathway was activated. Meanwhile, the increased expression of NRF1 confirmed that rhein was involved in the regulation of mitochondrial biogenesis. Hence, the activation of SIRT1/PGC-1α-mediated mitochondrial biogenesis by rhein might be a key mechanism for triggering the mitochondrial antioxidant defense system (Figure 8).

In summary, we used an AD-like neuronal oxidative stress model induced by Aβ_1-42_ oligomers to screen the antioxidant activities of the five anthraquinones, including rhein, emodin, aloe-emodin, chrysophanol, and physcion. According to the results of intracellular ROS and ΔΨm, rhein exhibited outstanding antioxidant activity and inhibited oxidative stress-associated neuronal apoptosis. More importantly, rhein activated mitochondrial biogenesis as an endogenous antioxidant defense system against Aβ_1-42_ oligomer-induced oxidative stress. That is to say, CytOx in the respiratory chain complex IV inhibited the production of ROS from electron leakage and SOD helped to eliminate superfluous ROS by promoted mitochondrial biogenesis. Western blot analysis further confirmed that the SIRT1/PGC-1α pathway activated by rhein was a potential antioxidant pathway involved. Taken together, our results provide evidence that rhein activates mitochondrial biogenesis regulated by the SIRT1/PGC-1α pathway as an antioxidant defense system against Aβ_1-42_ oligomer-induced oxidative stress. This study contributes to the fundamental research for the antioxidant therapy of AD.

## Data Availability

The original contributions presented in the study are included in the article/Supplementary Material, further inquiries can be directed to the corresponding authors.

## Abbreviations

ΔΨm, mitochondrial membrane potential; Aβ, β-amyloid; AD, Alzheimer’s disease; BCA, bicinchoninic acid; CD, circular dichroism; CytOx, cytochrome C oxidase; cyto c, cytochrome c; H2O2, hydrogen peroxide; mPTP, mitochondrial permeability transition pore; mtDNA, mitochondrial DNA; MTT, 3-(4,5-dimethylthiazol-2-yl)-2,5-diphenyltetrazolium bromide; NO, nitric oxide; NRF1, nuclear respiratory factor 1; PD, pharmacodynamic; PDB, Protein Data Bank; PGC-1α, peroxisome proliferator-activated receptor gamma coactivator 1-alpha; PI: propidium iodide; ROS, reactive oxygen species; SIRT1, sirtuin 1; SOD, superoxide dismutase; TEM, transmission electron microscopy; TFAM, transcription factor A; ThT, thioflavin T.
