# Supplementary material for: Rhein Relieves Oxidative Stress in an Aβ1-42 Oligomer-Burdened Neuron Model by Activating the SIRT1/PGC-1α-Regulated Mitochondrial Biogenesis
Source: Front Pharmacol. 2021 Sep 10;12:746711. doi: 10.3389/fphar.2021.746711 (PMC8461019; doi:10.3389/fphar.2021.746711)
Supplement: Supplementary file 1 [file DataSheet1.docx]

Supplementary Material

## Supplementary Figures


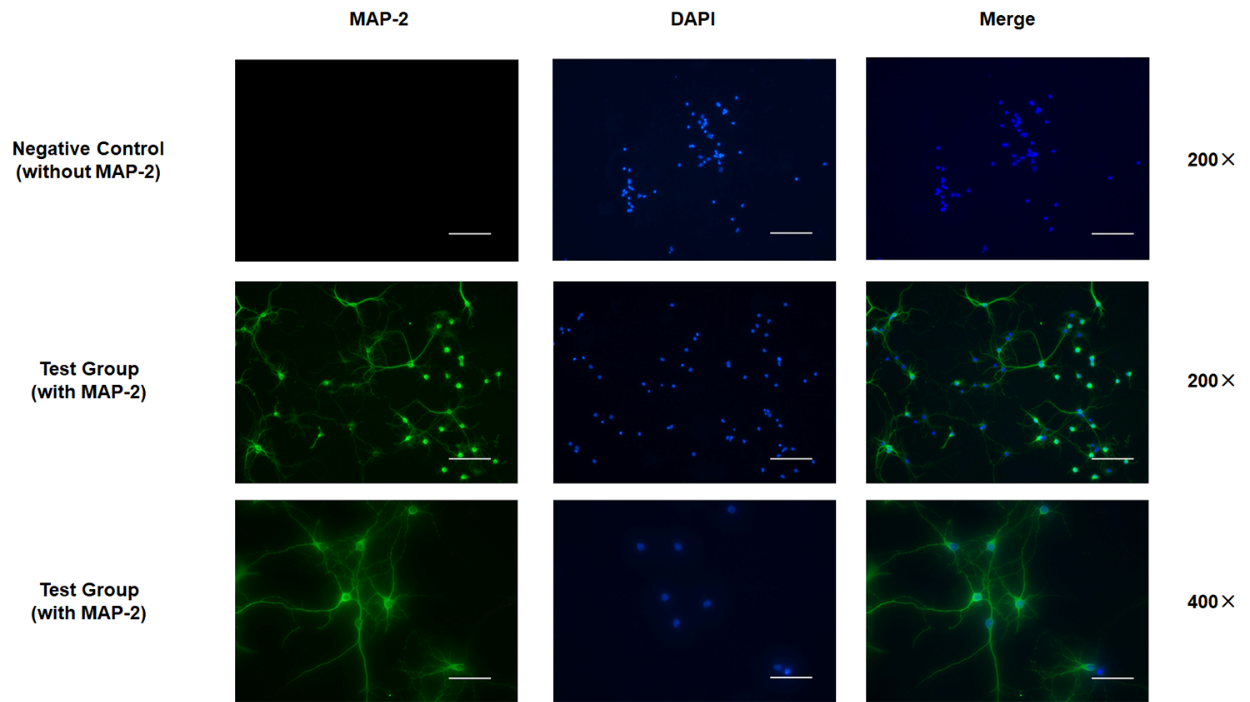


**Supplementary Figure 1.** The primary neurons were identified by the immunofluorescence assay. Negative Control: extracted and isolated cells from neonatal SD rats were incubated with DAPI instead of anti-MAP-2 antibodies. Test Group: extracted and isolated cells from neonatal SD rats were incubated with both anti-MAP-2 antibodies and DAPI. MAP-2: green, cytoskeleton protein in neurons; DAPI: blue, nucleus. Scale bars: 50 μm in 200×, 25 μm in 400×.


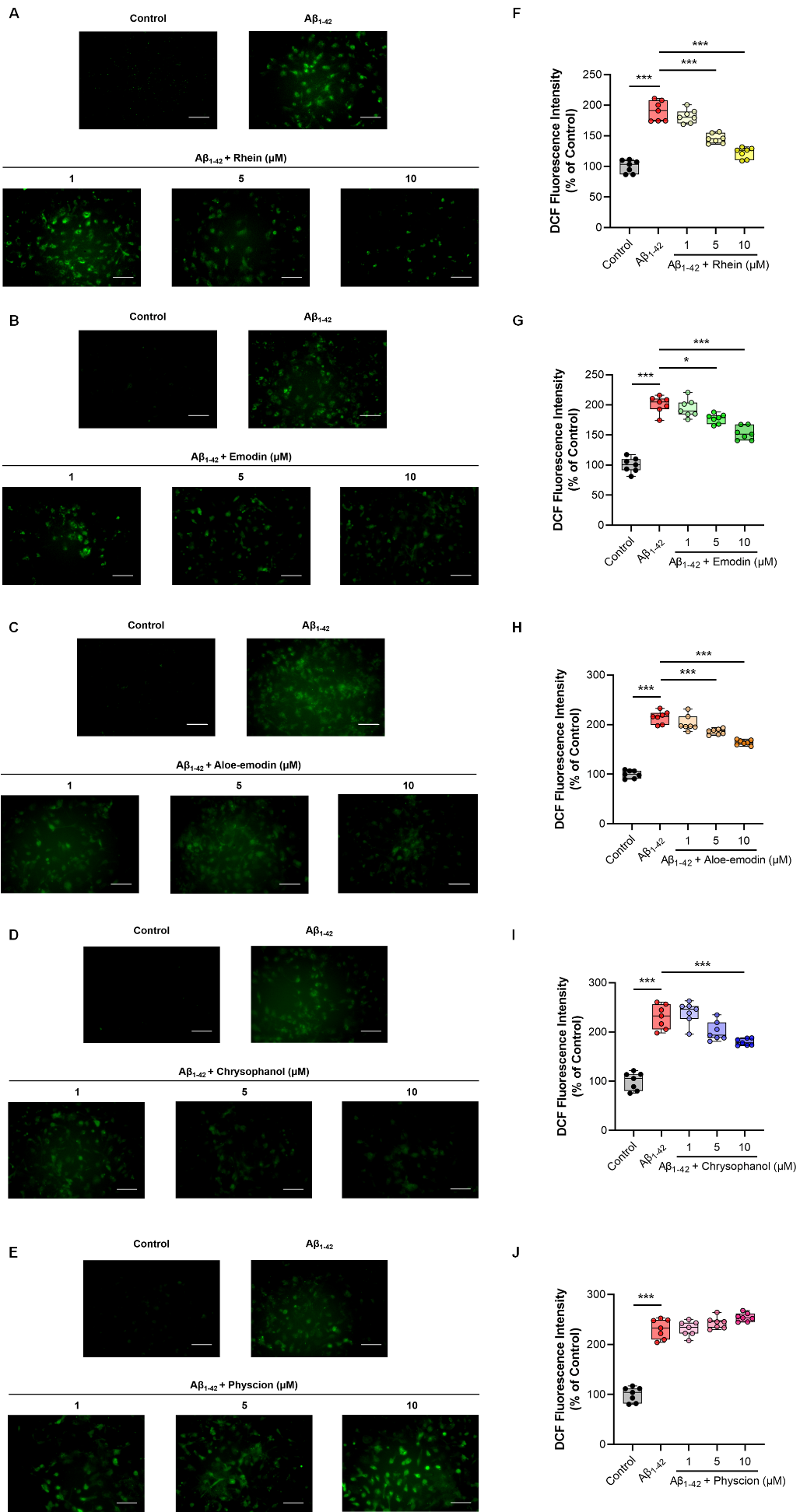


**Supplementary Figure 2.** The effects of the five anthraquinones on intracellular ROS level. Primary neurons were incubated with Aβ_1-42_ oligomers and each anthraquinones for 24 h at 37°C, respectively. Untreated primary neurons were the control group, and primary neurons treated with Aβ_1-42_ oligomers alone were the Aβ_1-42_ group. **(A-E)** Representative fluorescence images of intracellular ROS of primary neurons incubated with Aβ_1-42_ oligomers and rhein, emodin, aloe-emodin, chrysophanol, or physcion, respectively. Scale bars: 50 μm. **(F-J)** Quantification of intracellular ROS by the fluorescence intensity of DCF and normalization to the control group (*n* = 7). Data are presented as the mean ± standard deviation (SD). **p* < 0.05, ****p* < 0.001 compared with the Aβ_1-42_ group (one-way ANOVA).


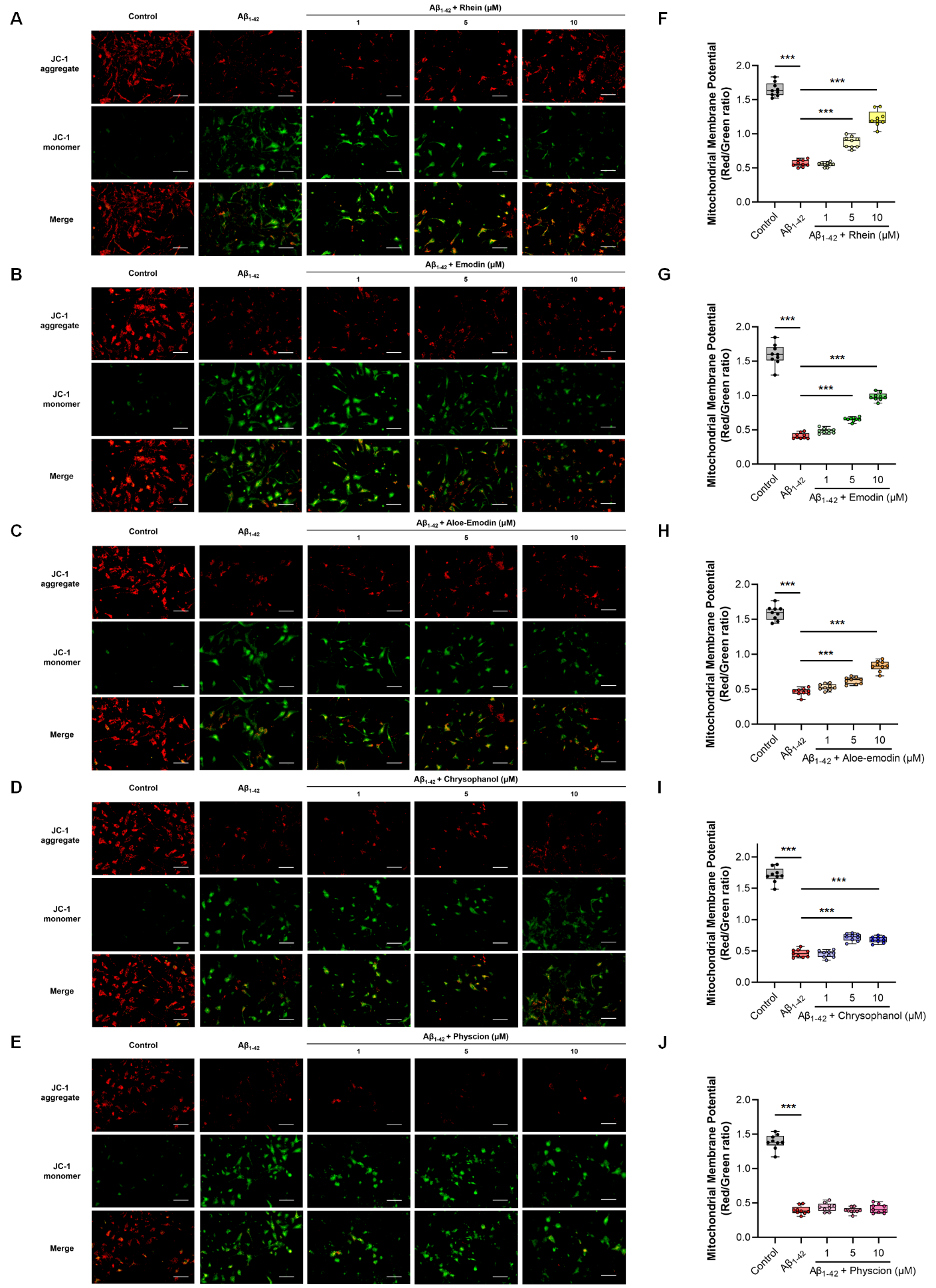


**Supplementary Figure 3.** The effects of the five anthraquinones on ΔΨm. Primary neurons were incubated with Aβ_1-42_ oligomers and each anthraquinones for 24 h at 37°C, respectively. Untreated primary neurons were the control group, and primary neurons treated with Aβ_1-42_ oligomers alone were the Aβ_1-42_ group. **(A-E)** Representative fluorescence images of ΔΨm of primary neurons incubated with Aβ_1-42_ oligomers and rhein, emodin, aloe-emodin, chrysophanol, or physcion, respectively. Scale bars: 50 μm. **(F-J)** Quantification analysis of ΔΨm by the fluorescence intensity of red/green ratio with JC-1 (*n* = 9). Data are presented as the mean ± standard deviation (SD). ****p* < 0.001 compared with the Aβ_1-42_ group (one-way ANOVA).
